# A Serosurvey of Multiple Pathogens in American Black Bears (*Ursus americanus*) in Pennsylvania, USA Indicates a Lack of Association with Sarcoptic Mange

**DOI:** 10.3390/vetsci6040075

**Published:** 2019-09-20

**Authors:** Kevin D. Niedringhaus, Justin D. Brown, Mark A. Ternent, Christopher A. Cleveland, Michael J. Yabsley

**Affiliations:** 1 College of Veterinary Medicine, Southeastern Cooperative Wildlife Disease Study, University of Georgia, Athens, GA 30602, USA; kevindn@uga.edu (K.D.N.); ccleve@uga.edu (C.A.C.); 2 Department of Veterinary and Biomedical Sciences, Pennsylvania State University, University Park, PA 16802, USA; jdb56@psu.edu; 3 Pennsylvania Game Commission, Harrisburg, PA 17110, USA; mternent@pa.gov; 4 Warnell School of Forestry and Natural Resources, University of Georgia, Athens, GA 30602, USA

**Keywords:** *Sarcoptes*, mange, black bear, *Trichinella*, *Toxoplasma*, canine adenovirus, canine distemper virus, parvovirus

## Abstract

Infectious diseases, particularly of wildlife, are intrinsically linked to human and domestic animal health. Reports of sarcoptic mange in black bears (*Ursus americanus*) are increasing in multiple states in the USA and while the reason is unknown, mange in other species has been associated with immunosuppression from multiple causes. Serum from bears across Pennsylvania were collected to determine the seroprevalence of five pathogens important for animal and/or human health: Canine distemper virus (CDV), canine parvovirus (CPV), canine adenovirus-1 (CAV), *Toxoplasma gondii*, and *Trichinella* sp. from bears with sarcoptic mange as well as bears that were clinically normal. Several of these pathogens, particularly canine distemper virus, are associated with immunosuppression and secondary infections in other hosts. In addition to describing the seroprevalence and relating these findings to data from other regions, statistics were performed to determine if antibodies to any of these pathogens were associated with mange in bears. The overall seroprevalence to these pathogens was as follows: CDV 7.1% (17/240), CPV 16% (15/94), CAV 6.9% (6/87), *Toxoplasma gondii* 64.9% (194/299), and *Trichinella* *spiralis* 3.2% (7/220). While there was no association between mange and antibodies to these pathogens, infection with one or more of these pathogens has implications for bears, other wildlife, domestic animal, and human health.

## 1. Introduction

Infectious diseases in wildlife are increasingly recognized as being linked to human and domestic animal health. The emergence and expansion of multiple diseases in wild animals, including chytridiomycosis, white nose syndrome, and chronic wasting disease, among others, can have significant welfare, economic, and conservation implications for companion animals, livestock, and other free-ranging wildlife [1,2,3,4]. Additionally, some of the most important emerging pathogens in humans are believed to have originated from wild animals [5]. As a result, it is increasingly important to continually monitor and study the presence of diseases and pathogens in free-ranging species. American black bears (*Ursus americanus*) are one of several wildlife species with an expanding population in many states in the eastern United States, and this expansion potentially affects the interface between this species and humans and domestic animals [6,7].

Sarcoptic mange, caused by the astigmatid mite *Sarcoptes scabiei*, is an important disease of wildlife and domestic animals, as well as in humans where the disease is known as scabies [8]. In humans, a severe form of scabies, known as crusted or Norwegian scabies, results in severe, typically non-pruritic hyperkeratosis and is considered to occur in patients that have a compromised immune system due to co-infections with immunosuppressive pathogens or a result of immunosuppressive therapy, among other reasons [9]. Sarcoptic mange in carnivores has many similarities to crusted scabies in humans and is often characterized by severe hyperkeratosis, alopecia, and loss of nutritional condition [10]. Variation in disease severity between individuals, regardless of the host, is likely multifactorial and involves the interaction of various risk factors including strain of mite involved, presence of toxins, co-infections, and host immune status [11,12,13,14]. Canine distemper virus (CDV) is a pathogen that commonly infects wildlife hosts and results in a primary fatal disease or results in immune suppression allowing for secondary infections [15]. The mechanisms that result in clinical disease and secondary infections in animals with CDV infection may also be important in the development of crusted scabies in humans. [15,16].

Sarcoptic mange is an emerging disease in American black bears and is now commonly reported in the Northeastern and Mid-Atlantic United States, particularly in Pennsylvania [17]. The cause of the increasing incidence and geographical expansion of this disease in bears over the last 30 years is unknown and likely multifactorial. Possible explanations include the emergence of a highly-virulent or bear-adapted mite strain, an increasing bear population encouraging the heightened transmission of mites, or a subclinical co-infection or environmental variable making bears more susceptible to clinical disease [18,19]. In addition to *S. scabiei*, black bears are commonly infected by a wide diversity of bacterial and viral pathogens and other parasites [20,21,22,23,24,25,26,27,28,29]. Infection and subsequent seroconversion from many of these pathogens is common, but clinical disease due to infectious pathogens, other than from *S. scabiei*, is considered rare in free-ranging black bears [26,30]. However, clinical disease from canine distemper virus, *Toxoplasma gondii*, and canine adenovirus-1 (CAV) in free-ranging bears has only recently been reported which has a potential temporal association with the significant increase in prevalence and distribution of clinical sarcoptic mange in bears in Pennsylvania, although expanding bear, dog, cat, or mesocarnivore populations could also have contributed to the recent reports of these diseases [17,31,32,33].

The objectives of this paper are (1) to provide serologic data on five common pathogens (CDV, CAV, canine parvovirus (CPV), *T. gondii*, and *Trichinella spiralis*) from black bears in Pennsylvania, USA; (2) compare the results with selected previous serology studies in black bears throughout the USA and Canada; and (3) to compare the presence of antibodies of these pathogens between bears that are clinically normal from those that have sarcoptic mange. We hypothesize that bears with antibodies against CDV, which is known to cause immunosuppression in other wildlife species, are more likely to have clinical sarcoptic mange compared to bears without antibodies to CDV [15,34].

## 2. Materials and Methods

**Figure 1:**
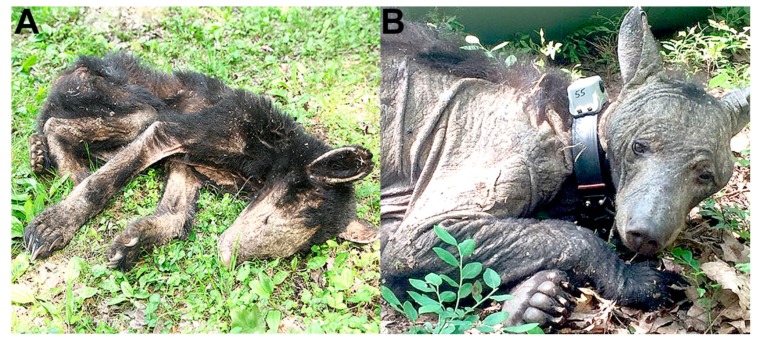
Black bears with sarcoptic mange. (**A**) Young black bear with clinical sarcoptic mange, note severe emaciation and hair loss. (**B**) Collared sow with severe alopecia as a result of sarcoptic mange.

**Figure 2:**
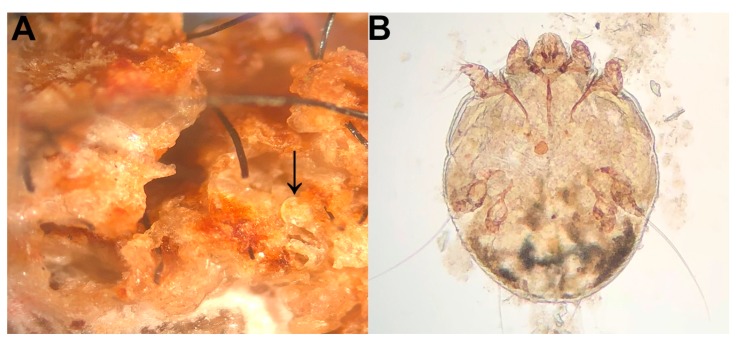
Microscopic views of *S. scabiei*. (**A**) Severe hyperkeratosis in the epidermis associated with round mites (arrow). (**B**) High-magnification image of an adult, female *S. scabiei* mite; this species can be differentiated from other mites on bears by its round shape and short legs.

Between 2014–2016, serum was collected from adult and yearling bears during den checks of radio-collared sows (February and March) and during routine trapping associated with ongoing bear population monitoring efforts in Pennsylvania. Blood was collected in serum separator tubes and kept cool in the field. Samples were centrifuged at the end of the day and serum was stored at −20 °C until testing was performed. All bears handled were thoroughly examined for any skin lesions. If skin lesions were observed (Figure 1), deep skin scrapes were collected into 70% ethanol and later examined microscopically to determine the presence of *S. scabiei* based on morphological criteria [8] (Figure 2). Skin scrapes were performed on all bears with skin lesions regardless of severity in an attempt to identify cases with less severe disease. All procedures complied with the University of Georgia’s Institutional Animal Care and Use Committee (IACUC; A2013-10-016 and A2015-05-13).

To determine the presence of antibodies to CAV and parvovirus, serum neutralization, and hemagglutination inhibition assays, respectively, were performed by the Athens Veterinary Diagnostic Laboratory in Athens, GA, USA as described [35,36]. Antibodies to CDV were detected using a serum neutralization assay as described [37]. Antibodies to *T. gondii* and *Trichinella* sp. were detected using a modified agglutination test and enzyme-linked immunosorbent assay (ELISA) (SafePath Laboratories, Carlsbad, CA, USA), respectively, at the United States Department of Agriculture Animal Parasitic Disease Laboratory, and these data were previously included in another publication [38,39]. Seropositive criteria were based on the following titers: CAV and CDV ≥ 4, CPV ≥ 10, and *T. gondii* ≥ 25. Criteria for *Trichinella* sp. included a corrected optical density value of >0.30.

To obtain other studies for comparison, a literature search was performed using all combinations of key words “black bear” and “ursus americanus” as well as “canine distemper”, “parvovirus”, “toxoplasma”, ”trichinella”, and “adenovirus” using Google scholar and Pubmed search engines. Chi-squared tests were performed to compare the proportion of bears with antibodies to each pathogen between those with clinical mange and those that were clinically normal with alpha = 0.05. Statistical analyses were performed using R, Version 3.0.1 (https://www.r-project.org) [40].

## 3. Results

Serum samples from 337 bears were included in this study, including 50 samples from bears with confirmed sarcoptic mange and 287 samples from bears with no evidence of mange (although not all bears were tested for all five pathogens due to limited sample availability for certain individuals). Antibody prevalence for each pathogen is summarized in Table 1. Of the five pathogens included, the prevalence of *T. gondii* was highest (194/299 bears, 64.9%) followed by parvovirus (15/94, 16%), whereas antibodies to *Trichinella* sp. and CAV were relatively uncommon being found in 7/220 (3.2%) and 6/87 (6.9%) bears, respectively. Canine distemper virus prevalence was 7.1% (17/240). Differences in antibody prevalence were not statistically different between mange and clinically-normal bears for all five pathogens. Table 2 shows the seroprevalence of the five tested pathogens in black bears from other studies.

## 4. Discussion

The potential role of co-infections in cases of clinical sarcoptic mange is incompletely understood in any animal or human host [61]. In this study, the presence of antibodies to several common pathogens in bears was not associated with sarcoptic mange. Of the pathogens investigated, CDV was most frequently associated with immunosuppression and secondary bacterial and parasitic infections in wildlife hosts [34]. The lack of association between CDV and mange in this study may be the result of bears being inherently susceptible to clinical disease after *S. scabiei* infestation regardless of their immune status. The seroprevalence of CDV in Pennsylvania bears in this study is within with the range of results from bears in other regions that are not experiencing increased reports of sarcoptic mange. Prevalence to CDV in black bears varied across previous studies and ranged from 0% in Alaska to over 30% in Maryland, USA [41,45].

The other four pathogens investigated are not commonly considered to cause significant immunosuppression in wildlife hosts. The lack of statistical association may reflect a true lack of association, the result of a relatively small sample size, or the antibody responses of these pathogens being altered as a result of mange. The seroprevalence of CPV and CAV in this study is similar to that described from bears in Maryland during a similar time period [45] and from bears from Florida in the 1990s [43]. Since bears are commonly exposed to these pathogens and clinical disease has not been reported from CPV infection in bears in Pennsylvania, it is presumed that these pathogens are unlikely to be a significant health concern for bears in this region, but the ability of bears to amplify or shed these pathogens is unknown. It is plausible that the expansion of black bears in eastern USA could result in increased contact with domestic animals resulting in a higher frequency of transmission of these pathogens (as well as *S. scabiei*). Additionally, it is unknown whether antibodies to CPV detected in this study were from presumed CPV-2 or from other closely-related parvoviruses.

Clinical disease due to CDV, CAV, and *T. gondii*, in free-ranging bears has only recently been reported [31,32,33]. The first published clinical case of canine distemper in a black bear was from a yearling in Pennsylvania in 2011. This animal displayed clinical signs and had lesions consistent with canine distemper in other species [31]. In addition, two other previously unpublished cases of canine distemper have been recently detected in Pennsylvania [62]. One case involved a cub that was found dead in a den with a clinically normal sow in 2015. This cub had bronchointerstitial pneumonia with rare syncytia and intracytoplasmic inclusion bodies consistent with CDV. CDV was also detected in the lung by fluorescent antibody testing. The second case was suspected based on inclusion bodies in the brain but was not confirmed via additional testing.

Disease from CAV infection was reported in multiple captive black bears as well as in a free-ranging brown bear in Alaska [33,63,64,65]. In addition to clinical disease, the seroprevalence of CAV may be increasing in bears across North America. There is evidence that CAV has been endemic in Alaska for 40 years or more and that seroprevalence, as well as brown bear cub mortality, is increasing in this region [41,66,67]. As a result, and due to the severe implications of this pathogen on the health of domestic dogs, it is important to continue to monitor for changes in CAV seroprevalence in bears (both clinically and subclinically).

The published case of toxoplasmosis in a black bear was from New Jersey and involved a cub with multi-organ necrosis associated with *T. gondii* [32]. No known clinical disease has occurred due to canine or feline parvovirus or *Trichinella* sp. infection despite the detection of antibodies from prior years [26,30,68,69]. Despite the widespread seroconversion of bears in Pennsylvania to *T. gondii*, there was no evidence of antibodies to *T. gondii* being a risk factor for mange. While the seroprevalence in this study is less than in previous studies from bears in Pennsylvania (Table 2), the overall trend appears to be the increasing seroprevalence in bears from other populations over time as well as a higher seroprevalence in bears in eastern North America compared to western North America, but more research and statistical analyses on this subject are warranted. Additionally, variations in seroprevalence may be reflected in the variability of the assay used as well as the age of the bear as seroprevalence appears to increase in black bears in Pennsylvania with age [55,56]. Emerging seroprevalence and the first case report of mortality from *T. gondii* warrants heightened surveillance of this pathogen due to wildlife and domestic animal implications [32].

The assays and titers used as diagnostic cutoffs, when described, varied between many studies further complicating comparisons between regions and over time. Assays for CDV included one or a combination of ELISAs and indirect fluorescent antibody assays as well as serum neutralizations with cutoff values from serum neutralization ranging from 1 in a study from Alaska [48] to 12 in the Northwest Territories, Canada [46]. Indirect fluorescent antibody assays were used for CPV in a study from California [28], and positive cutoff titers for studies also using hemagglutination inhibition assays, similar to this study, ranged from 20 in all other studies to 10 in the current study. Serum neutralization assays were used exclusively for CAV and while most studies used a cutoff titer of ≥4, one study used ≥10 as positive, possibly resulting in a lower seroprevalence [42]. The assays used to detect antibodies to *T. gondii* included modified hemagglutination tests, latex agglutination tests, indirect hemagglutination assay, and Sabin–Feldman dye tests. When modified agglutination tests, as used in this study, were employed, the diagnostic cutoffs ranged from 16 in a study in the Appalachian Mountains, USA [59] to 64 in Florida, USA [43]. The assays for *Trichinella* sp. included the latex particle test, inhibition agglutination assay, as well as ELISA as for this study. Corrected optical densities that were considered positive were most often 0.3 in other studies as well as in the current study [28,41,60].

This study also emphasizes that bears in Pennsylvania were commonly exposed to three zoonotic parasites: *S. scabiei*, *T. gondii*, and *Trichinella* sp. While there is only anecdotal evidence suggesting mild classical scabies can occur in humans handling bears with clinical sarcoptic mange [70], reports of transmission and subsequent disease have been documented in humans after contracting mites from other animal hosts, although humans in these situations are considered ‘dead end’ hosts due to the lack of mite replication and subsequent transmission [71,72,73,74]. Consumption of bear meat has been suggested as a rare cause of toxoplasmosis in humans but is currently considered to be the greatest risk of trichinellosis in humans [75,76,77]. Appropriately cooking bear meat to kill these two parasites is considered one of the best ways to mitigate risks [57,78,79].

To our knowledge, this study is the first to investigate seroprevalence to common black bear pathogens in Pennsylvania including CDV, CAV, and CPV as well as the first to explore the potential role of co-infections in an emerging disease (sarcoptic mange) in black bears. Additional studies are warranted to further explore the emergence of sarcoptic mange in black bears in Northeastern United States and any potential risk factors within the affected populations. Understanding the mechanisms of transmission, variation in host immune responses, and overall *S. scabiei* exposure can help our understanding of this emerging disease.

## Figures and Tables

**Table 1:** Comparison of the seroprevalence of five pathogens in black bears from Pennsylvania between groups of bears with confirmed sarcoptic mange and clinically normal bears.

Pathogen	Mange No. Pos/Tested (%)	Non-Mange No. Pos/Tested (%)	Total No. Pos/Tested (%)	X^2 †^	*p*
CDV	2/31 (6.5)	15/209 (7.2)	17/240 (7.1)	0.0216	0.883
CPV	9/46 (19.6)	6/48 (12.5)	15/94 (16.0)	0.8743	0.350
CAV	4/46 (8.7)	2/41 (4.9)	6/87 (6.9)	0.4920	0.483
*T. gondii*	22/32 (68.8)	172/267 (66.4)	194/299 (64.9)	0.2352	0.628
*Trichinella* sp.	0/32 (0)	7/188 (3.7)	7/220 (3.2)	1.2	0.267

CDV: Canine distemper virus; CPV: Canine parvovirus; CAV: Canine adenovirus; ^†^ Chi-squared test: Estimation of the difference between the expected and observed values.

**Table 2:** Previous studies showing the seroprevalence of select pathogens infecting free-ranging black bears in North America compared to the current study.

Years Sampled	Number Positive/No. Tested (%)	Location	Reference
**CDV**			
N/A	0/47 (0)	Great Smoky Mt. NP, USA	[20]
1988–1991	0/76 (0%)	Alaska, USA	[41]
1993–1997	8/165 (4.8%)	Northwestern states, USA	[42]
1993–1995	5/66 (8%)	Florida, USA	[43]
1994–2001	1/38 (3%)	Banff NP and BC, Canada	[44]
1999–2011	25/82 (30.5%)	Maryland, USA	[45]
2001–2003	24/157 (15.3%)	California, USA	[28]
2002–2010	2/6 (33%)	Northwest Territories, Canada	[46]
2014–2016	17/240 (7.1%)	Pennsylvania, USA	Current Study
**CPV**			
1988–1991	0/76 (0%)	Alaska, USA	[41]
1993–1995	10/62 (16%)	Florida, USA	[43]
1999–2011	10/82 (12.2%)	Maryland, USA	[45]
2001–2003	1/157 (0.6%)	California, USA	[28]
2002–2010	0/14 (0%)	Northwest Territories, Canada	[46]
2014–2016	15/94 (16%)	Pennsylvania, USA	Current Study
**CAV**			
1984	1/33 (3%)	Washington, USA	[47]
1988–1991	3/76 (4%)	Alaska, USA	[41]
1993–1995	4/66 (6%)	Florida, USA	[43]
1993–1997	3/165 (1.8%)	Northwestern States, USA	[42]
1994–2001	8/38 (8%)	Alberta /British Columbia, Canada	[44]
1999–2011	7/82 (8.5%)	Maryland, USA	[45]
2014–2016	6/87 (6.9%)	Pennsylvania, USA	Current Study
***T. gondii***			
N/A	40/149 (27%)	California, USA	[48]
N/A	132/328 (40.2%)	New Jersey, USA	[49]
N/A	1/3 (33%)	Ontario, Canada	[50]
1971–1977	23/303 (8%)	Idaho, USA	[51]
1971–1974	7/16 (43.8%)	Ontario, Canada	[52]
1976–1996	62/143 (43%)	Alaska, USA	[53]
1988–1991	6/40 (15%)	Alaska, USA	[54]
1989–1992	532/665 (80%)	Pennsylvania, USA	[55]
1993	22/28 (78.6%)	Pennsylvania, USA	[56]
1993–1995	37/66 (56%)	Florida, USA	[43]
1993–1997	89/198 (45%)	Northwestern States, USA	[42]
1994–2001	5/38 (13%)	Banff NP and BC, Canada	[44]
1996–1997	120/143 (84%)	North Carolina, USA	[57]
1999–2011	70/82 (85.4%)	Maryland, USA	[45]
2001–2003	67/239 (28%)	California, USA	[28]
2002–2010	2/16 (12.5%)	Northwest Territories, Canada	[46]
2004–2006	13/29 (44.8%)	Florida, USA	[58]
2012–2013	33/53 (62%)	Central Appalachia, USA	[59]
2014–2016	194/299 (64.9%)	Pennsylvania, USA	Current Study
***Trichinella* spp.**			
N/A	18/141(13%)	California, USA	[48]
1971–1977	16/122 (13%)	Idaho, USA	[51]
1988–1991	11/76 (14.5%)	Alaska, USA	[41]
1993–1997	2/103 (1.9%)	Oregon, USA	[60]
1996–1997	0/79 (0%)	North Carolina, USA	[57]
2001–2003	6/80 (7.5%)	California, USA	[28]
2014–2016	7/220 (3.2%)	Pennsylvania, USA	Current Study

CDV: Canine distemper virus; CPV: Canine parvovirus; CAV: Canine adenovirus.
